# S100B Protein but Not 3-Nitrotyrosine Positively Correlates with Plasma Ammonia in Patients with Inherited Hyperammonemias: A New Promising Diagnostic Tool?

**DOI:** 10.3390/jcm12062411

**Published:** 2023-03-21

**Authors:** Anna Maria Czarnecka, Marta Obara-Michlewska, Dorota Wesół-Kucharska, Milena Greczan, Magdalena Kaczor, Janusz Książyk, Dariusz Rokicki, Magdalena Zielińska

**Affiliations:** 1 Department of Neurotoxicology, Mossakowski Medical Research Institute Polish Academy of Sciences, Pawińskiego 5, 02-106 Warsaw, Poland; 2 Department of Pediatrics, Nutrition, and Metabolic Diseases, Children’s Memorial Health Institute, Al. Dzieci Polskich 20, 04-730 Warsaw, Poland

**Keywords:** ammonia, urea cycle disorders, S100B, 3-nitrotyrosine

## Abstract

Individuals with inherited hyperammonemias often present developmental and intellectual deficiencies which are likely to be exaggerated by hyperammonemia episodes in long-term outcomes. In order to find a new, systemic marker common to the course of congenital hyperammonemias, we decided to measure the plasma level of S100 calcium-binding protein B (S100B), which is associated with cerebral impairment. Further, we analyzed three mechanistically diverged but linked with oxidative–nitrosative stress biochemical parameters: 3-nitrotyrosine (3-NT), a measure of plasma proteins’ nitration; advanced oxidation protein products (AOPP), a measure of protein oxidation; and glutathione peroxidase (GPx) activity, a measure of anti-oxidative enzymatic capacity. The plasma biomarkers listed above were determined for the first time in congenital hyperammonemia. Also, the level of pro- and anti-inflammatory mediators (i.e., IL-12, IL-6, IL-8, TNF-α, IL-1β, and IL-10) and chemokines (IP-10, MCP-1, MIG, and RANTES) were quantified. S100B was positively correlated with plasma ammonia level, while noticeable levels of circulating 3-NT in some of the patients’ plasma did not correlate with ammonia concentration. Overall, the linear correlation between ammonia and S100B but not standard oxidative stress-related markers offers a unique perspective for the future identification and monitoring of neurological deficits risk-linked with hyperammonemia episodes in patients with inherited hyperammonemias. The S100B measure may support the development of therapeutic targets and clinical monitoring in these disorders.

## 1. Introduction

Ammonia, an end product of amino acids and protein metabolism, exerts neurotoxic effects when present in excess. In order to reduce this excess, ammonia is detoxified via the urea cycle, which consists of a chain of enzymatic reactions localized in periportal hepatocytes that parallels glutamine synthesis in the brain [1,2,3].

Mutations in any of the genes constituting the urea cycle, encoding six enzymes and two mitochondrial transporters, lead to urea cycle disorders (UCDs), termed ‘primary hyperammonemias’. The most common UCD is an X-linked deficiency in ornithine transcarbamylase (OTC), whereas autosomally recessive inherited deficiency in N-acetyl glutamate synthase (NAGS), carbamoyl synthase (CPS1), argininosuccinate synthetase (ASS), argininosuccinate lyase (ASL), or arginase 1 (AR1) are less frequent [4]. Secondary hyperammonemias accompany metabolic disorders, i.e., organic acidurias, where the urea cycle malfunction is a consequence of enzymatic aberrations disabling the synthesis of the urea cycle substrates [4,5]. The most representative of these are organic acidemias, e.g., methylmalonic, propionic, and isovaleric acidemia, wherein the hyperammonemia is a result of the inhibition of NAGS and/or CPS1 by intermediary metabolites (e.g., methylmalonic or propionic acid, isovalerylglycine) accumulating due to the enzymatic defect [4].

The neurotoxic effect of ammonia can result in growth retardation, psychomotor delay, and neurological impairment [6,7,8]. Importantly, repeated episodes of hyperammonemia might potentiate neurological dysfunction with an eventual fatal outcome if untreated [5,9]. The aforementioned situation is especially true for neonatal-onset diseases, wherein patients’ outcomes are uniformly poor, even with relatively early recognition and treatment [10,11,12]. However, the symptoms of hyperammonemia are often not specific enough to instantly point to it as a reason for a neurological decline. Furthermore, the specificity and validity of measured ammonium concentrations are often limited [13,14].

In order to find a new, systemic marker common to the course of congenital hyperammonemias, we decided, for the first time, to measure the plasma concentration of S100 calcium-binding protein B (S100B). Importantly, an S100B increase was previously found in astrocytes in vitro upon ammonia treatment [15] and in patients with hepatic encephalopathy (HE), mechanistically related to ammonia accumulation [16,17,18,19]. Promisingly, the S100B protein was reported as a peripherally detectable brain injury biomarker in acute brain injury, neuroinflammatory, neurodegenerative, and psychiatric disorders [20,21,22,23].

The molecular mechanisms of ammonia neurotoxicity include overlapping processes involving oxidative–nitrosative stress and inflammation pathways, as it has been frequently documented in preclinical and clinical studies of hyperammonemia-related HE [24], though it escaped detailed investigation in congenital hyperammonemias. Thus, we investigated whether markers of oxidative-nitrosative stress and inflammatory cascades are associated with the course of the abovementioned disease entities. Notably, the nitrosative stress-modified end-point metabolite, 3-nitrotyrosine (3-NT), the proposed biomarker of subclinical HE in cirrhotic patients, was measured. Furthermore, the oxidative stress-indicative markers, such as glutathione peroxidase (GPx) activity and advanced oxidation protein products (AOPP), were measured. Likewise, we assessed the levels of inflammatory cytokines and chemokines in plasma samples obtained from patients diagnosed with congenital hyperammonemias, specifically primary (ornithine transcarbamylase deficiency, citrullinemia type 1 and 2) and secondary (hyperinsulinism-hyperammonemia, methylmalonic acidemia, and propionic acidemia).

## 2. Materials and Methods

### 2.1. Patient Characteristics

Eleven patients with diagnosed inborn errors of metabolism, classified as primary or secondary hyperammonemia, were included in the analysis (Table 1). Standard analytical measurements, including metabolites pattern, C-reactive protein (CRP) level, and liver enzyme activity, were conducted along with clinical verification of neurological status (Table 1). Patients remained under the care of the Department of Pediatrics, Nutrition, and Metabolic Diseases, Children’s Memorial Health Institute, Warsaw. All of the procedures followed were in accordance with the ethical standards of the responsible committee on human experimentation (institutional and national) and with the Helsinki Declaration of 1975, as revised in 2000. Informed written consent was obtained from all patients being included in the study or from their legal guardians. The study was approved by the Committee for Medical Research Ethics of the Children’s Memorial Health Institute in Warsaw (No: 41/KBE/2018).

Five of the patients had ornithine transcarbamylase deficiency (OTCD). Three patients (Patients 1–3), who had a late-onset OTCD form, came from one family, each carrying the same mutation: c.622G>A in *OTC*. Patient 4 carries a c.802A>G mutation, also presenting late OTCD onset, whereas Patient 5 developed with early onset (at 4 weeks of age). At the moment of sampling, Patients 1–4 were metabolically compensated.

Patient 6 suffers from methylmalonic aciduria (MMA) type mut0. The first symptoms appeared early, on the 3rd day of life, in the form of acute decompensation. Starting at 1.5 months of life, the patient was fed by PEG. Despite treatment with diet or temporarily with benzoate, ammonia was constantly elevated, up to 200 µg/mL. Normalization of ammonia was achieved later on with carglumic acid.

Patient 7, 14 years of age, developed symptoms on the 6th day of life, followed by a diagnosis of propionic acidemia (PA). From the 3rd year of age, the patient was fed by PEG. Despite diet and benzoate treatment, the patient presented constant hyperammonemia (124–356 µmol/L).

Patient 8 suffered from PA. The first symptoms appeared at the 6th month of age as acute metabolic decompensation. Hyperammonemia was constant (up to 197 µg/mL) to the moment of carglumic acid introduction, which allowed for ammonia normalization. 

Patient 9 was admitted at the age of 2 months because of neonatal cholestasis and liver insufficiency. Ammonia was elevated in the range of 109–261 µg/mL. NGS analysis revealed c.1453–2A>T variant in *SLC25A13*–citrin deficiency. Hypertransaminasemia and mild hyperammonemia persisted until the introduction of a low-carbohydrate diet.

Patient 10 underwent severe metabolic decompensation with brain edema from the 2nd day of life, with a maximal ammonia concentration of 1316 µg/mL, persisting until sodium butyrate administration. 

In Patient 11, genetic investigations demonstrated *de novo* mutation in *GLUD1* (c.1493C>T). Patient’s elevated ammonia concentration did not decrease with diazoxide or upon consumption of a diet with leucine restriction and did not cause symptoms of decompensation.

### 2.2. Samples

All of the blood was collected at a single time point: at the time of the patient’s hospital admission. Plasma samples were collected from whole blood after centrifugation and aliquots were kept frozen at −80 °C until analysis.

### 2.3. Biochemical Analysis

All patients were evaluated by routine laboratory tests, including liver functions, complete blood count, c-reactive protein (CRP), and plasma ammonia.

The plasma concentration of certain amino acids—glutamate (GLU), glutamine (GLN), citrulline (CIT), ornithine (ORN), and arginine (ARG)—was determined by ion exchange chromatography followed by photometric detection after ninhydrine derivatization using the AminoTac JLC-500/V amino acid analyzer (JEOL, Tokyo, Japan).

#### 2.3.1. Inflammatory Cytokines and Chemokines

Selected inflammatory cytokines in plasma were determined using the Cytometric Bead Array Human Inflammatory Cytokines kit (#551811, BD Biosciences, Becton and Dickinson, San Diego, CA, USA), following the manufacturer’s instructions. The kit is designated for quantitative and simultaneous measurement of interleukin (IL)-6, IL-8, IL-10, IL-12, and tumor necrosis factor-alpha (TNF-α). The BD CBA Human Chemokine Kit was used to evaluate selected chemokine levels (pg/mL) (552990, BD Biosciences, Becton and Dickinson, San Diego, CA, USA). The kit is designated for the quantitative and simultaneous measurement of IL-8, RANTES, monokine induced by interferon γ (CXCL9/MIG), monocyte chemoattractant protein-1 (CCL2/MCP-1), and interferon γ-induced protein-10 (CXCL10/IP-10). Data acquisition (300 events for each cytokine and chemokine) was performed using a BD FACSCanto II flow cytometer with BD FACSDiva Software and FCAP Array software, version 3.0 (BD Biosciences, San Jose, CA, USA).

#### 2.3.2. 3-Nitrotyrosine

The plasma 3-nitrotyrosine (3-NT) was determined with the use of the OxiSelect™ Nitrotyrosine ELISA Kit (STA-305, Cell Biolabs Inc., San Diego, CA, USA). The procedure was based on a competitive enzyme-linked immunosorbent assay. Quantitative determination of 3-NT in plasma samples was performed in duplicates according to the manufacturer’s instructions. Briefly, 50 μL of plasma sample was added to each well of 96-well plates and was then shaken for 10 min on an orbital shaker. Fifty microliters of anti-tyrosine antibody were then added and incubated for one hour on an orbital shaker at room temperature. Then, each well was washed thoroughly three times with 250 μL wash buffer. After removing all wash buffer, 100 μL secondary antibody–enzyme conjugate was added to all wells and incubated for one hour at room temperature on an orbital shaker. Again, each well was washed thrice with 1× wash buffer. Substrate solution (100 μL) was added to each well and incubated for about 15 min, and as color developed, 100 μL of stop solution was added per well. The plate was read at 450 nm on a spectrophotometer using a microplate reader (SPECTROstar Nano, BMG Labtech, Ortenberg, Germany). A calibration curve was performed using nitrated bovine serum albumin, corresponding to 3-NT concentrations of 1.95–8000 nM. The kit has a nitrotyrosine-detection sensitivity range of 20 nM to 8.0 µM.

#### 2.3.3. Advanced Oxidation Protein Products (AOPP)

Advanced oxidation protein product (AOPP) levels were analyzed using the OxiSelect AOPP kit (STA-318, Cell Biolabs, San Diego, CA, USA) following the manufacturer’s protocol. Ten microliters of plasma, with a 200 µL final volume, were subjected to 10 µL of chloramine reaction initiator followed by 20 µL of stop solution. Absorbance was recorded at 340 nm using the spectrophotometric microplate reader (SPECTROstar Nano, BMG Labtech, Ortenberg, Germany).

#### 2.3.4. Glutathione Peroxidase Activity Assay

GPx activity was analyzed using the Glutathione Peroxidase Assay Kit (ab102530, Abcam, Cambridge, UK), according to the manufacturer’s instructions. In brief, plasma samples were depleted of all GSSG by incubating the sample with glutathione reductase (GR) and reduced glutathione (GSH) for 15 min. GPx activity was determined by adding cumene hydroperoxide and incubating for 0 and 5 min. The absorbance was determined at OD340. One unit of GPx activity was defined as the amount of enzyme that causes the oxidation of 1 µmol of NADPH to NADP+ under the assay kit conditions per minute at 25 °C.

#### 2.3.5. S100 Calcium-Binding Protein B (S100B)

According to the manufacturer’s recommendations, the S100B protein concentration in plasma was measured using the EZHS100B-33K kit from Millipore (Millipore, Billerica, MA, USA). In brief, the assay is a sandwich ELISA based, sequentially, on the capture of S100B molecules from samples in the wells of a microtiter plate coated by a pre-titered amount of anti-S100B monoclonal antibody; the binding of biotinylated anti-S100B polyclonal antibody to the captured molecules; the conjugation of horseradish peroxidase to the immobilized biotinylated antibodies; and the quantification of immobilized antibody–enzyme conjugates by monitoring horseradish peroxidase activity in the presence of the substrate 3,3′,5,5′-tetramethylbenzidine. The enzyme activity was measured spectrophotometrically by the increased absorbency at 450 nm (SPECTROstar Nano, BMG Labtech, Ortenberg, Germany), corrected from the absorbency at 590 nm after the acidification of formed products. The increase in absorbency is directly proportional to the amount of captured S100B in the unknown sample, derived by interpolation from a reference curve with standards of known concentrations of S100B. The kit has an S100B detection sensitivity range of 2.7–2000 pg/mL. All samples were analyzed in duplicate, and the average of the two was used for statistical analysis.

### 2.4. Statistics

Statistical analysis was performed using GraphPad Prism 7.0 (GraphPad Software Inc., La Jolla, CA, USA). Linear regression analysis was performed in order to identify correlations between ammonia levels and other biochemical parameters. The solid line is the result of linear regression, showing the corresponding 95% confidence bands of the line of best fit and the Pearson correlation coefficient (R2). All analyses were performed using a significance level of 0.05.

## 3. Results

Parameters related to the clinical characteristics and applied treatment are shown in Table 1. Most of the pediatric patients (except Patients 5 and 9) presented neurological impairment, which could manifest in the form of delayed development (defined for individuals < 5 years of age), intellectual disability (defined for individuals > 5 years of age), or epilepsy. Also, one of the four adult patients (Patient 4) manifested symptoms of intellectual disability and psychiatric disturbances. The morphology of blood cells and the CRP protein level (0.04–0.33 mg/dL) remained within the normal range and did not indicate an ongoing infection in any of the included patients. The ASPAT and ALAT values were also within the reference range (26–55 and 24–78 U/L, respectively). A plasma ammonia level < 80 μg/mL was regarded as the norm, and in 7 patients (Patients 5 to 11), an increased level was observed (95–364 μg/mL). The plasma amino acid (GLN, GLU, CIT, ORN, and ARG) levels, assessed for a discriminative diagnosis of metabolic diseases, were within the normal range, except for increased CIT in Patient 9 and Patient 10 and increased ORN and ARG in Patient 10.

**Figure 1:**
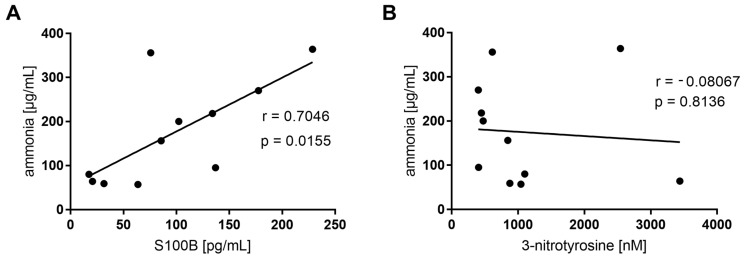
Pearson r correlation of ammonia and S100B (**A**) and 3-NT (**B**) among patients (n = 11).

The clinical outcome data for S100B, 3-NT, AOPP concentration, and GPx activity in plasma are presented in Table 2. A linear correlation (r = 0.705, considered significant when *p* < 0.05) of S100B protein (17.48–228 pg/mL range) with plasma ammonia concentration (57–364 μg/mL) was revealed (Figure 1A). Furthermore, 3-NT levels, ranging from 401.61 to 3439.41 nM, were elevated substantially in some patients but were unrelated to ammonia levels (Figure 1B).

The concentrations of inflammatory cytokines and chemokines measured in plasma are presented in Table 3. The levels of cytokines (IL-12, TNF-α, IL-10, IL-6, IL-1β, and IL-8) and chemokines (IP-10, MCP-1, and MIG) were consistently low, except for Patients 5 and 6, who presented substantially increased levels of IL-12, IL-10, IL-6, IL-1β, IL-8, IP-10, and MCP-1 (Patient 5) or TNF-α, IP-10, MCP-1, and MIG (Patient 6). Contrary to other cytokines and chemokines, the absolute values of RANTES concentrations are characterized by relatively high variability within the group (964.85–54,423.4 pg/mL). Although the measured levels of selected cytokines for some patients varied markedly within the group, a characteristic pattern cannot be discerned. None of the cytokines and chemokines studied correlated with either the ammonia levels or the type of congenital hyperammonemia. 

**Figure 2:**
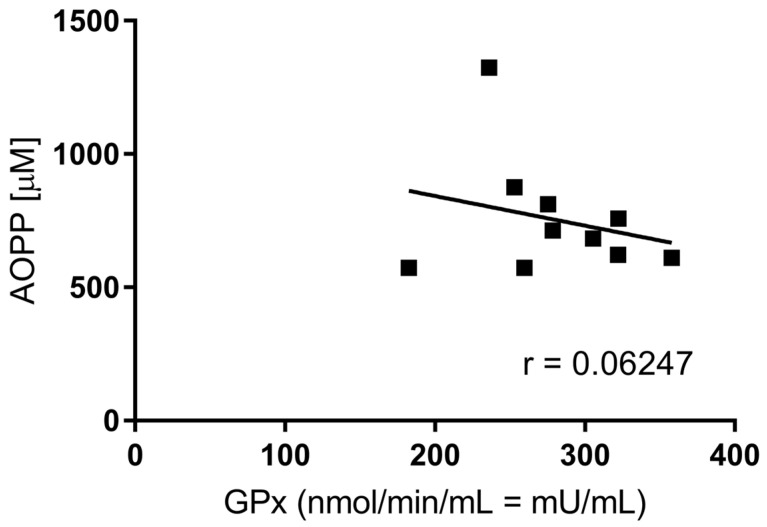
Pearson r correlation of AOPP level and GPx activity among patients (n = 11).

There were neither significant increases in AOPP level (572.79–1324.14 μM range within the group) nor decreases in GPx activity (182.51–357.88 mU/mL) nor a significant correlation between the two parameters in the studied group of patients. However, a trend can be observed toward an inverse correlation between pro- and anti-oxidative markers’ concentrations and activity, AOPP, and GPx (Figure 2).

## 4. Discussion

In the present study, we proposed two circulating metabolites, S100B and 3-NT, that may have diagnostic utility in patients with congenital hyperammonemias. We hypothesized that the determination of selected metabolites, along with the measurement of ammonia concentration in plasma, biochemical hallmarks defining hyperammonemia episode occurrence, may serve as circulating indicators of neurological decline and systemic oxidative–nitrosative stress, respectively. The study documented two key findings: (i) a positive correlation between ammonia and S100B levels; (ii) distinctive levels of 3-NT, uncorrelated to ammonia in the plasma of studied patients.

Plasma samples were obtained from individuals diagnosed and followed at the Children’s Memorial Health Institute due to several congenital hyperammonemias. The occurrence of clinically and biochemically verified episodes of hyperammonemia in the cohort of 11 previously recruited and genetically characterized patients was documented as described in Section 2.

According to the guidelines for pediatric hyperammonemia diagnosis, ammonia measurement, despite technical pitfalls, is consistently recommended as a discriminating factor for further and more specific, i.e., metabolic and genetic, evaluation. However, accurate assessment of plasma ammonia levels is challenging, since the final ammonia concentration is affected by many factors, including the site of blood specimen collection, the handling of the specimen, and the analytical method used [25]. It has been reported that no UCDs patient having had >300 μmol/L initially or >480 μmol/L peak plasma ammonia exhibited normal psychomotor development, underscoring the potential utility of new markers in terms of the neurological status of pediatric patients with congenital hyperammonemias [11]. It was also shown that ammonia concentrations may correlate with disease severity and predict mortality correlated with encephalopathy resolution [26,27]. In our study, the ammonia concentrations of recruited patients presented significantly elevated but variable values, and the correlative analysis did not fully cover patients’ neurological status. However, it is essential to note that in most patients, noticeable neurological deteriorations were observed and verified in the clinical evaluation (Table 1).

Particular neuromarkers are derived from specialized cell types within the brain, e.g., neuron-specific enolase (NSE) present in neurons, S100B in astrocytes, and myelin basic protein in oligodendrocytes. It has to be acknowledged that many brain-derived proteins can escape into the peripheral circulation despite an intact blood–brain barrier (BBB) [28,29,30]. BBB, limits their size and quantity, determines the peripheral values indicating cell injury, and the severity of BBB impairment [31]. The S100B protein is stable and relatively unaffected by storage, changes in temperature, and freeze–thaw cycles. In contrast to NSE, it is not affected by hemolysis in the sample [32]. Unlike other brain biomarkers such as GFAP or NSE, S100B release is independent of cell death [32]. Presuming the above, measuring S100B plasma concentration offers a unique opportunity to associate patients’ clinical/neurological status with the routine measurement of analytes.

S100B is an acidic, homodimeric protein present physiologically at low or undetectable concentrations in serum, and elevated S100B serum levels have been detected in several neuropathological conditions [33]. Significantly, its extracellular concentration determines its neuroprotective (nanomolar) or detrimental (micromolar) effects, respectively [34], the latter by inducing apoptosis, stimulating the release of proinflammatory cytokines or nitric oxide from astroglial cells, and contributing to oxidative stress [35,36,37,38]. It is worth noting that S100B exhibits features that are considered to be typical of inflammatory molecules, such as the activation of microglia or the stimulation of IL-6 and TNF-α secretion [34,39,40].

In our study, the concentration of the peripheral biomarker of the central nervous system (CNS) impairment, S100B protein, demonstrated a positive correlation with plasma ammonia levels (*p* < 0.05). To the best of our knowledge, no previous studies have investigated the possible correlation between S100B and ammonia in children with an inborn error of metabolism. Thus, our results, for the first time, support S100B determination as a promising non-invasive diagnostic tool for inherited hyperammonemias.

Previously, it was demonstrated that serum S100B and IL-6 levels were associated with HE in pediatric patients suffering from acute liver failure, indicating that measuring these markers may be of benefit to the assessment of neurological injury, impacting clinical decisions [41]. There was no significant correlation between HE and a biomarker for neuronal injury, NSE. Similarly, another study demonstrated an S100B increase in a group of cirrhotic adults with stage 1 and 2 HE, while NSE was unaltered [33]. Elevated S100B has also been shown in patients with fulminant hepatic failure [42]. It was also demonstrated that high ammonia levels induce an increase in S100B release without alterations to its intracellular content [15]. Acute exposure to ammonia in astrocytes increases cell calcium [43]; therefore, this mechanism could be involved in S100B release induced by ammonia.

A few limitations may temper the promise of S100B as a viable diagnostic and prognostic biomarker. S100B concentrations fluctuate at several developmental stages [44,45,46]. Bouvier et al. mapped out reference ranges of the serum levels of S100B from a large cohort of healthy children [47]. The higher concentrations of S100B found in children under three years old could be related to the permeability of the BBB, low renal excretion of S100B, and reflect the dynamic neurodevelopmental processes [47]. The baseline concentrations seemed to stabilize after the age of 20 years [31] and decrease with age [48]. Therefore, it is crucial to establish reference values in studies involving the measurement of blood S100B in pediatric and adolescent patients.

We excluded S100B originating from the cells of non-neural tissues (i.e., adipocytes, chondrocytes, and bone marrow cells) [32] as not crucial in our study. The extensive study of 200 subjects revealed that extracranial sources of S100B do not affect serum levels in intact subjects [49]. Therefore, the diagnostic value of S100B and its negative predictive value in neurological diseases appear not to be compromised in the clinical setting [49].

Whether S100B increase may additionally exacerbate or compensate for CNS impairment in congenital hyperammonemias should be investigated. Since serum S100B levels may increase before neuronal cell death or a significant change in neurological function [49], it should also be confirmed whether it has predictive value for the progression and severity of underlying neurological impairment during an episode of metabolic decompensation.

As was mentioned above, S100B features are typical of inflammatory molecules. We subsequently measured a standard systemic inflammation-related panel of cytokines and chemokines in this context. However, neither significant changes in the levels of pro-inflammatory cytokines and chemokines nor correlation with ammonia or patients’ clinical status and the type of congenital hyperammonemia were observed. In the studied group of patients, no changes in CRP levels were noted, and infections were ruled out by clinical assessment and blood tests.

A significant correlation was observed between CRP levels and inflammatory gene markers in patients with subclinical HE [50], but lack of correlation was documented elsewhere [51]. Importantly, parallel measurements of CRP and interleukin 6 (Il-6) turned out to be insufficient to establish inflammation, as concluded from large-scale epidemiological studies and meta-analyses of mortality outcomes due to a variety of causes, including cancer, cardiovascular disease, and metabolic syndrome (e.g., [52,53,54]). Consequently, assays of more unambiguous inflammatory biomarkers (e.g., IL-1β and TNF-α), were suggested to be necessary [55]. Of note, pro- and anti-inflammatory cytokines, but not CRP, were inversely correlated with severity and neurological assessments in major depression [56].

The second marker utility verified in this study was the 3-NT, an oxidative–nitrosative stress end-product. For the first time, to our knowledge, 3-NT was measured in patients with congenital hyperammonemias. Our study failed to demonstrate a positive correlation between ammonia and 3-NT levels. However, and it should be clearly emphasized here, the concentration of 3-NT varied considerably between patients, with some reaching high levels within a studied group. Protein nitration is a common process that occurs under physiological conditions, and the conversion of tyrosine to 3-NT can have a deleterious effect on protein function, resulting in cell damage [57,58]. The determination of 3-NT concentration correlated with psychometric tests was used to identify patients with subclinical HE [59,60]. Additionally, a significant increase in 3-NT levels in protein has been associated with a wide range of diseases, such as asthma [61], cardiovascular diseases, diabetes mellitus [62], diseases associated with immunological reactions [63], neurological diseases, and psychiatric disorders [64]. Our results cannot unquestionably support the 3-NT predictive value; thus, measurement should be extended to a larger and more homogenous group of patients and be secured with validated reference values which, to our knowledge, are unknown for pediatric patients.

Since the redox-based mechanism is associated with a variety of pathological events, including metabolic disorders with inborn hyperammonemias [65,66] and, direct measurement of reactive oxygen species levels with accuracy and precision is difficult due to their short lifespan and high reactivity, we employed further approaches to measure advanced oxidation protein products (AOPP) and glutathione peroxidase GPx, well-documented indicators of oxidative stress. To our knowledge, there has been no attempt to analyze this product in the plasma of congenital hyperammonemia patients, and AOPPs with advanced glycated end products were typically elevated in patients with renal complications, atherosclerosis, or diabetes mellitus [67,68]. The results documented unaltered plasma AOPP levels in the studied group. However, a negative result, cannot definitively rule out the AOPP value to determine the effects of oxidative stress. In the studied group of patients, there was also no marked decrease in GPx, nor a significant correlation with ammonia level. Since the previous estimation of reduced glutathione (GSH) levels in UCDs patients was proposed as a useful indicator of oxidative stress in line with considerably reduced GSH levels [69], the small sample size in our study limits both GPx and AOPPs’ conclusive value, and its utility as an oxidative stress indicator in studied patients should be extended in our future research.

## 5. Conclusions

The management of congenital hyperammonemia is complicated by the lack of currently applied biomarkers offering clear predictive value. Assessing plasma ammonia level is challenging, and measurement might be affected by many factors; thus, the determination of plasma ammonia and S100B may serve as an additional tool in monitoring the neurological deficits risk-linked with hyperammonemia episodes in patients with inherited hyperammonemias. It is also advisable for future research to measure the 3-NT concentration in the peripheral bloodstream and verify whether it correlates with neurological decline. Nonetheless, it should be confirmed whether a proposed metabolite can also have a predictive value for the progression and severity of underlying neurological impairment during acute decompensation.

## Figures and Tables

**Table 1:** Clinical characteristics of patients ^1^.

**Patient’s ID**	**Diagnosis**	**Age [Years]**		**Gender**		**Clinical Phenotype**					**Treatment**		
						**Hepatic**	**Neurologic Description**		**Asymptomatic**		**Drugs**	**Diet**	
1	OTCD	43		M					x		no	normal	
2	OTCD	37		M					x		no	normal	
3	OTCD	34		M					x		no		
4	OTCD	18		M			x (intellectual disability, psychiatric symptoms)				arginine	protein-restricted diet	
5	OTCD	0.5		F					x		arginine	normal	
6	MMA	1.5		M			x (delayed development)				no	protein-restricted diet	
7	PA	14		F			x (intellectual disability, epilepsy)				sodium benzoate	protein-restricted diet	
8	PA	11		F			x (intellectual disability, epilepsy)				sodium benzoate	protein-restricted diet	
9	citrullinemia type 2	0.2		M		x					no	normal	
10	citrullinemia type 1	1.1		F			x (delayed development, epilepsy)				sodium benzoate	protein-restricted diet	
11	Hi-HA	1		M			x (epilepsy)				no	normal	
**Patient’s ID**	**Plasma amino acids**					**Blood morphology**					**CRP** **(mg/mL)**	**ALT ASPAP U/L**	
	**GLU** **(0–88)**	**GLN** **(415–964)**	**CIT** **(12–55)**	**ORN** **(30–106)**	**ARG** **(36–145)**	**WBC (10^3^/µL)**	**RBC (10^6^/µL)**	**HCT (%)**	**HGB (g/dL)**	**PLT (10^3^/µL)**	**<0.5**		
1	-	-	-	-	-	-	-	-	-	-	-	-	-
2	48.4	605.5	12.1	73.3	79.1	-	-	-	-	-	-	-	-
3	37.6	684.3	15.5	51.5	49.6	-	-	-	-	-	-	-	-
4	30.4	718.4	16.3	61.4	52.6	6.1 (4–10)	5.01 (4.1–6.2)	44 (40–54)	15.5 (14–18)	203 (150–450)	-	24	35
5	63.2	739.5	9.5	88.4	60.7	8.2 (4–20)	4.79 (3.8–5)	33 (33–39)	11.1 (10–13)	479 (140–350)	<0.03	33	38
6	63.1	396.9	46.8	47.1	95.4	9.4 (4.5–13)	4.91 (4.3–5.5)	35 (34–41)	12.6 (10.9–14.2)	385 (140–350)	0.03	55	78
7	45.3	432.7	19.5	40.5	57.5	2.1 (4–10)	3.48 (3.7–5.1)	29 (37–47)	9.6 (12–16)	196 (150–0400)	0.13	31	35
8	45.4	425.6	29.1	45.3	67.3	4.2 (4–12)	4.43 (4.5–5.5)	36 (37–43)	12.4 (12–15.5)	229 (150–400)	0.04	19	24
9	13.5	215.4	429.9	120	242.1	11.5 (4–20)	2.74 (3.8–5)	22 (30–37)	7.7 (9.5–13)	442 (150–0350)	0.33	17	39
10	64.2	743.4	2456.9	18.3	23.7	14.6 (4.5–13)	5.04 (4.3–5.5)	41 (34–41)	14 (10.9–14.2)	527 (150–350)	-	6	30
11	53.5	635.7	30.4	83.3	171.6	7.6 (4.5–13)	4.48 (4.3–5.5)	35 (34–41)	12.3 (10.9–14.2)	289 (150–350)	0.03	19	26

^1^ Abbreviations: ARG, arginine; CIT, citrulline; F, female; GLN, glutamine; GLU, glutamate; Hi-HA, hyperinsulinism–hyperammonemia; M, male; MMA, methylmalonic acidemia; PA, propionic academia; ORN, ornithine; OTCD, ornithine transcarbamylase deficiency.

**Table 2:** S100B, 3-nitrotyrosine, AOPP concentration, and GPx activity in plasma ^1^.

Patient’s ID	Diagnosis	Ammonia [μg/mL]	S100B [pg/mL]	Nitrotyrosine [nM]	GPx [nmol/min/mL = mU/mL]	AOPP [µM]
1	OTCD	59	31.61	875.68	272.31	-
2	OTCD	80	17.48	1101.21	322.34	758.38
3	OTCD	57	63.67	1043.51	322.12	621.44
4	OTCD	64	20.8	3439.41	305.40	683.60
5	OTCD	95	136.99	405.11	252.92	875.50
6	MMA	156	85.63	845.80	278.66	713.33
7	PA	356	75.71	611.32	357.88	610.63
8	PA	200	102.31	473.63	236.21	1324.14
9	Citrullinemia type 2	218	134.13	448.03	259.61	572.79
10	Citrullinemia type 1	364	228.67	2542.68	275.43	811.53
11	Hi-Ha	270	177.61	401.61	182.51	572.79

^1^ Abbreviations: AOPP, advanced oxidation protein products; GPx, glutathione peroxidase activity; Hi-HA, hyperinsulinism–hyperammonemia; MMA, methylmalonic acidemia; PA, propionic academia; OTCD, ornithine transcarbamylase deficiency; S100B, S100 calcium binding protein B; -, no data.

**Table 3:** S100B, 3-nitrotyrosine, AOPP concentration, and GPx activity in plasma ^1^.

Patient’s ID	Diagnosis	Cytokines [pg/mL]						Chemokines [pg/mL]			
		IL-12	TNF-α	IL-10	IL-6	IL-1β	IL-8	IP-10	MCP-1	MIG	RANTES
1	OTCD	0.73	1.13	0.95	1.73	0.24	5.67	94.66	6.38	93.56	17,391.49
2	OTCD	0.87	0.40	1.26	0.56	0.00	5.44	98.51	15.17	70.89	15,962.86
3	OTCD	0.70	0.53	0.99	1.89	0.11	1.59	174.02	19.17	72.39	20,809.61
4	OTCD	1.21	0.00	1.49	1.79	0.48	4.23	101.07	24.68	113.22	6968.73
5	OTCD	3.25	0.58	36.21	1345.01	6.26	56.57	1521.87	166.34	1101.97	1473.47
6	MMA	1.26	1.17	1.86	3.31	0.07	11.16	513.42	428.54	961.02	4997.10
7	PA	0.87	0.84	0.59	1.53	0.33	1.92	140.40	23.69	93.05	1229.31
8	PA	0.07	0.11	0.86	0.83	0.37	9.76	105.25	44.83	113.50	4927.25
9	citrullinemia type 2	0.44	0.59	1.78	96.52	0.10	76.41	128.34	64.34	251.04	964.85
10	citrullinemia type 1	1.07	0.13	1.56	2.07	0.00	9.22	103.23	47.34	92.63	54,423.48
11	HiHa	1.26	0.33	1.68	0.15	0.00	8.05	120.72	97.63	219.62	22,575.80

^1^ Abbreviations: AOPP, advanced oxidation protein products; GPx, glutathione peroxidase activity; HiHA, hyperinsulinism–hyperammonemia; MMA, methylmalonic acidemia; PA, propionic academia; OTCD, ornithine transcarbamylase deficiency; S100B, S100 calcium binding protein B; -, no data.

## Data Availability

Data are not available due to privacy or ethical restrictions.
